# Clinical experience of bench surgery combined with autotransplantation after three-dimensional laparoscopic nephrectomy for the treatment of highly complex renal tumor

**DOI:** 10.1186/s12957-023-03246-9

**Published:** 2023-11-29

**Authors:** Yangkai Xu, Jiawen Huang, Xiaodong Fan, Zhichao Wang, Jiangyong Lou, Xiaoming Liu, Guobin Weng

**Affiliations:** Department of Urology, Ningbo Urology and Nephrology Hospital, Ningbo, 315100 Zhejiang China

**Keywords:** Autotransplantation of the kidney, Bench surgery, Renal cell carcinoma, 3D laparoscopic nephrectomy

## Abstract

**Objective:**

To assess the feasibility and safety of three-dimensional (3D) laparoscopic nephrectomy in combination with bench surgery and autotransplantation for treating highly complex renal tumors.

**Materials and methods:**

The clinical data of six patients with highly complex renal cell carcinoma were collected. All patients underwent 3D laparoscopic nephrectomy in combination with bench surgery and autotransplantation by the same surgeons, two of them had previously undergone laparoscopic partial nephrectomy for contralateral renal cancer.

**Results:**

The total operative time was 366 ± 65 min, the warm ischemia time (WIT) was 1.3 ± 0.4 min, and the cold ischemia time was 121 ± 26 min. While one patient received a diluted autologous blood transfusion, the intraoperative blood loss was 217 ± 194 ml. No increase in the serum creatinine (SCr) level was observed at postoperative day 30 compared with the preoperative time, and none of the patients received dialysis either during the hospital stay or to date. Although one patient underwent nephrectomy due to tumor recurrence in the transplanted kidney, the others reported no tumor recurrence or distant metastases on imaging to date.

**Conclusion:**

3D laparoscopic nephrectomy, when combined with bench surgery and autotransplantation, can become a feasible option for treating highly complex renal cell carcinoma cases when expecting to preserve renal function maximally.

**Supplementary Information:**

The online version contains supplementary material available at 10.1186/s12957-023-03246-9.

## Introduction

Renal cell carcinoma is among the ten most common cancers in men, and its incidence is steadily rising over the years [1]. Nephron-sparing surgery (NSS) is now being widely used as a recommended surgical modality for renal tumor cases, especially cT1-stage renal tumor patients, for it can preserve renal function to a great extent while completely removing the tumor and thus reducing the postoperative renal function decline [2–4]. However, in complex tumors with [(R)adius, (E)xophytic/endophytic properties, (N)earness to the collecting system or sinus, (A)nterior (a)/posterior (p) and the (L)ocation] (R.E.N.A.L) Nephrometry score ≥ 9, renal hilar mass, huge kidney tumor (tumor size > 7 cm), or tumors in a solitary kidney, NSS application is still debatable owing to its limitations of warm ischemia time, the requirement of complete tumor resection and wound suturing; these limitations increase the risk of positive incisal margin, postoperative urinary fistula, and postoperative bleeding [5–7]. According to the AUA guidelines [8], RN is recommended for complex renal tumors with a size > 7 cm. However, RN results in significant loss of normal kidney tissue and exerts adverse effects on preoperative kidney function. These occurrences increase the risk of kidney failure and subsequent dialysis postoperatively, thereby affecting patients’ quality of life [9, 10]. Thus, bench surgery combined with autotransplantation has been developed for preserving kidney function. In this procedure, after the excision, the kidney is subjected to external low temperatures for protection while performing lesion excision and tissue trimming. Furthermore, autologous kidney transplantation is performed to restore the kidney to its original position or pelvic fossa to achieve complete lesion resection while preserving the renal function profoundly. This procedure was first reported by Hardy in 1963 [11]. Currently, the main indications for this intervention are renal aneurysm, long-segment ureteral stenosis, renal arteriogenic hypertension, and complex renal malignancy [12]. Therefore, many centers have adopted ex vivo NSS in combination with autotransplantation, which helps in tumor removal and preserves maximum renal function for achieving good results. Although there are still drawbacks like greater surgical trauma and longer operation time, this combination offers a viable option for patients with highly complex renal tumors [13, 14].

The advancement of laparoscopic techniques has significantly contributed to the reduction of surgical trauma caused by nephrectomy [15]. Compared with the expensive cost of robot-assisted nephrectomy, laparoscopic nephrectomy is cheaper and can be widely used in developing countries such as China [16]. Compared with the traditional 2D laparoscopic system, the 3D laparoscopic intervention provides better intraoperative imaging effects and a smoother learning curve for the surgeon [17]. Although the 3D laparoscopic nephrectomy in combination with bench surgery and autotransplantation has been used for hilar renal artery aneurysm cases, it has not been utilized much for treating highly complex renal tumors [18]. Thus, this study aimed to summarize the clinical experience of six patients with highly complex renal tumors treated by 3D laparoscopic nephrectomy when combined with bench surgery and autotransplantation in our center.

## Materials and methods

### Clinical data

**Fig. 1:**
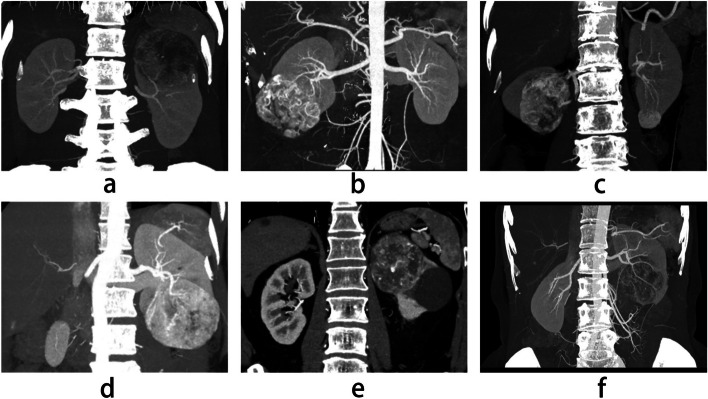
Computerized tomography scans of six patients (**a** to **f** shows patients 1 to 6)

The clinical data of six patients (3 men and 3 women, aged 32–59 years) who underwent 3D laparoscopic nephrectomy when combined with bench surgery and autotransplantation by the same surgeons at Ningbo Urology and Nephrology Hospital between November 2019 and December 2022 were collected, including body mass index (BMI), tumor size, preoperative 24-h urine volume, preoperative mean arterial pressure (MAP), preoperative SCr levels and so on (Table 1). The surgeons had more than 15 years of experience in kidney transplantation and renal tumor surgeries. Since two patients reported bilateral renal tumors simultaneously, they had undergone laparoscopic partial nephrectomy on the contralateral kidney 1 month before for diagnosis of clear cell carcinoma. Preoperative imaging examinations, including chest radiography, computed tomography (CT), and abdominal ultrasonography, were performed to confirm the absence of local infiltration and distant metastasis. Furthermore, preoperative renal computed tomography angiography (CTA, Fig. 1) was performed to assess the location and number of renal arteries and the complexities of the surgery. This study was approved by the Medical Ethics Committee of Ningbo Urology and Nephrology Hospital, and all patients signed informed consent forms.

**Table 1:** Preoperative clinical data of patients

Clinical data		Case identification number						Mean ± SD
		1	2	3	4	5	6	
Age, years		32	38	56	47	59	45	46 ± 10
BMI, kg/m^2^		18.6	20.1	25.3	21.9	23.6	22.1	21.9 ± 2.4
Gender		Male	Male	Female	Female	Male	Female	–
Tumor size, cm	Complex side	7.0	6.5	6.5	7.6	7.5	6.0	6.9 ± 0.6
	Contralateral	–	1.6	2.5	–	–	–	2.1 ± 0.6
RENAL score	Complex side	9P	10P	10P	10A	9P	9H	–
	Contralateral	–	4A	4A	–	–	–	–
Preoperative Scr, µmol/L		50	81	60	61	59	56	61 ± 10
Preoperative 24 h urine volume^a^, ml		850	1700	1650	1800	1300	1700	1500 ± 362
Preoperative MAP^b^, mmHg		83	77	88	87	106	85	88 ± 10
eGFR, mL/min/1.73 m^2^		136	106	98	104	105	108	110 ± 13
Operation date		2020/12/17	2020/12/10	2020/9/25	2019/11/14	2019/11/11	2021/3/5	–

^a^Preoperative 24 h urine volume was recorded as the 24-h urine volume on the day before operation day

^b^Preoperative MAP was measured on the morning of the operation day (MAP = 1/3arterial systolic pressure + 2/3arterial diastolic pressure)

### Surgical intervention

#### Three-dimensional laparoscopic nephrectomy

The patient was placed in the oblique supine position after anesthesia. After sterilization and draping, a paraumbilical pneumoperitoneum was created after inserting a 3D laparoscopic lens. Two surgical channels were further created inferior to the costal margin and 2 cm above the umbilicus, respectively, at the ipsilateral anterior axillary line.

The kidney’s anterior side was mobilized through Toldt’s gap between the peritoneal plane and Gerota’s fascia, while its posterior side was mobilized outside Gerota’s fascia. After finding the renal pedicle by following the gonadal vessels, the renal artery and vein, as well as the ureter, were dissected distally to the abdominal aorta and common iliac artery, respectively. All the above transections were performed after applying Hemo-o-locks to the clamp (see Additional file 1). Furthermore, the specimens were extracted from the Gibson incision on the corresponding side, a perinephric drainage tube was left in place, and the incision was temporarily sutured.

#### Workbench surgery and autotransplantation

The specimen was immediately inserted into the ice-cold perfusion fluid. After the removal of the perirenal fat, the kidney mass was removed, keeping the pseudocapsule as the boundary. Furthermore, the kidney defect was sutured in two layers with 3–0 absorbable and 2–0 barbed sutures. Consequently, the renal vessels and the ureter were trimmed to facilitate autotransplantation.

**Fig. 2:**
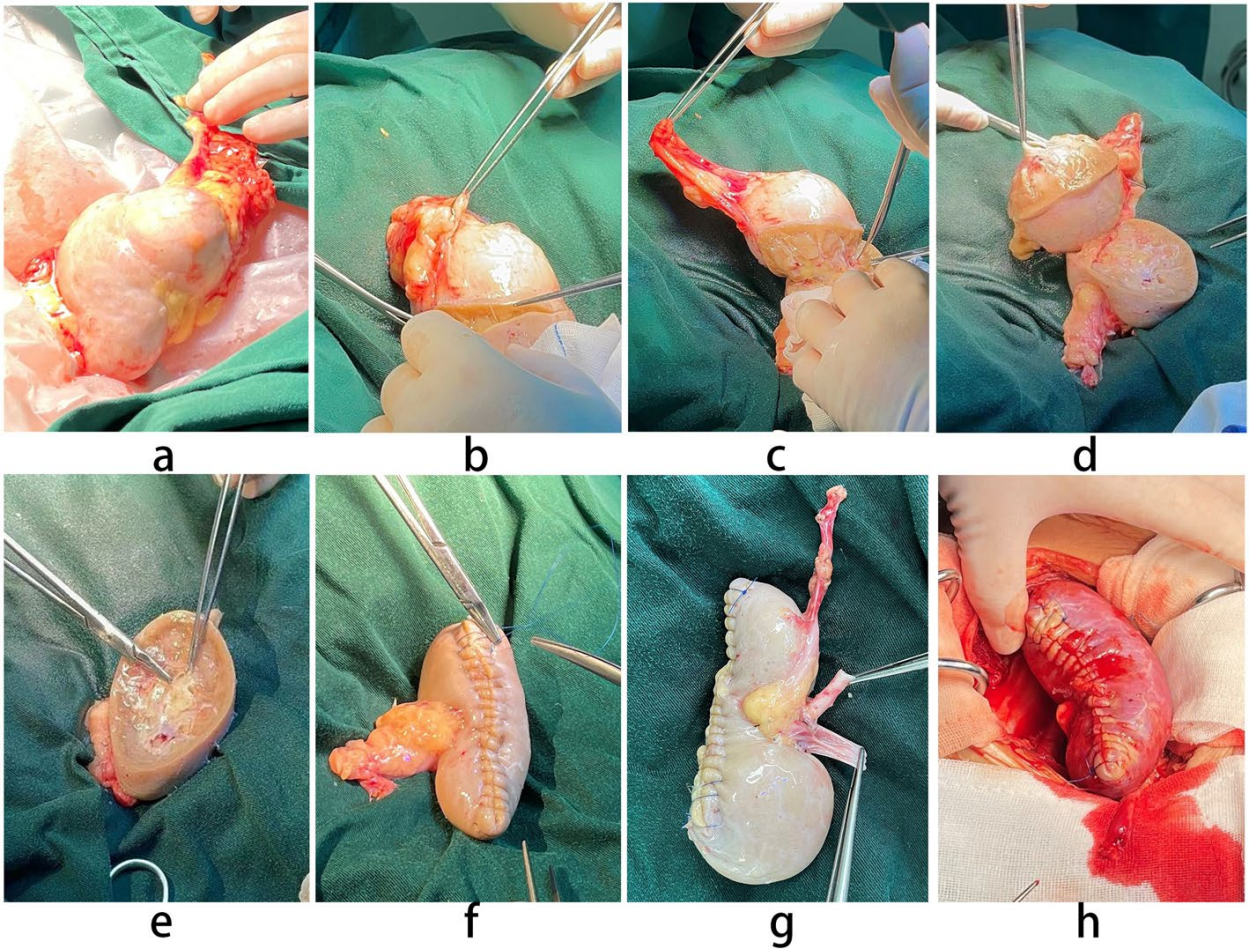
Complete removal of the renal mass while maximizing normal parenchyma preservation by bench surgery (**a**–**g**). No serious bleeding and ischemia in the autotransplanted kidney after restoring vascularity (**h**)

After repositioning the patient in the supine position, the Gibson incision was reopened, and the underlying tissue was dissected for access to the iliac fossa. The renal vessels were anastomosed in end-to-end and end-to-side directions to the internal iliac artery and external iliac vein, respectively. The blood flow was subsequently restored to check the renal parenchyma perfusion. After confirming the absence of severe bleeding and ischemia, the ureter was implanted into the lateral bladder wall, and a double-J tube was placed into the ureter as a stent (Fig. 2). Additionally, a drain was placed in the transplanted renal fossa before incision closure.

### Follow-up strategy

During postoperative hospitalization, patients’ SCr levels, 24-h urine volume, and MAP were measured regularly. After discharge, SCr level measurement, whole abdomen, and chest CT scan, as well as an ultrasound of the transplanted kidney, were performed routinely to evaluate renal function, tumor prognosis, and postoperative recovery.

### Statistical analysis

SPSS21.0 statistical software was used for data analysis. The data are expressed as the mean ± SD, and the valid numbers of mean ± SD is harmonized with the corresponding original data.

## Results

### Intraoperative condition

All patients underwent surgery as per the study protocol. The total operative time, warm ischemia time (WIT), and cold ischemia time were 366 ± 65 min, 1.3 ± 0.4 min, and 121 ± 26 min, respectively. Except for one patient who received an autologous blood transfusion, the other patients did not receive any form of blood transfusion, and the intraoperative blood loss was 217 ± 194 ml. There were no intraoperative complications except for intraoperative bleeding in one patient. The pathology analysis showed that all complex side tumors were Fuhrman grade I–III clear cell carcinomas without any positive surgical margin (Table 2).

**Table 2:** Intraoperative and histopathological data of patients

Clinical data	Case identification number						Mean ± SD
	1	2	3	4	5	6	
Warm ischemia, min	1.0	1.0	2.0	1.5	1.0	1.0	1.3 ± 0.4
Cold ischemia, min	93	147	126	88	121	150	121 ± 26
Operative time, min	300	442	426	325	400	300	366 ± 65
Intraoperative blood loss, ml	100	200	100	600	200	100	217 ± 194
Pathological type^a^	KIRC	KIRC	KIRC	KIRC	KIRC	KIRC	–
Fuhrman grade	1	2	1	2	3	1	–
Intraoperative complications	–	–	–	Bleeding	–	–	–

^a^*KIRC* kidney clear renal carcinoma

### Postoperative condition

The mean 24-h urine volume on the first postoperative day was 1965 ± 532 ml, which showed an increase compared with the preoperative values. The MAP on the first day after operation was basically the same as that before operation, and the MAP of all patients was relatively stable during the postoperative period in the hospital. The SCr level increased to varying degrees in all the patients on the first postoperative day, which then decreased over time. However, there was no apparent increase in the SCr levels on postoperative day 30 compared to the preoperative period, none of the patients received dialysis either during the hospital stay or to date. The main postoperative complications were urinary tract infections (4/6) and postoperative pain (3/6), which were relieved after symptomatic treatment with antibiotics and analgesics, respectively. Although one patient underwent nephrectomy due to tumor recurrence in the transplanted kidney as the diagnosis was clear cell carcinoma, other patients had no tumor recurrence or distant metastases on imaging till now. The ultrasound examination showed that the pulsatility index (PI) and resistance index (RI) of the segmental renal artery were favorable at 1 month after surgery, being 1.17 ± 0.22 and 0.66 ± 0.08, respectively. The mean postoperative hospital stay time was 12 ± 2 days, and the mean cost of hospital stay was 46724 ± 5915 yuan (Table 3).

**Table 3:** Postoperative clinical data of patients

Clinical data	Case identification number						Mean ± SD
	1	2	3	4	5	6	
Postoperative complications^a^	UTI, pain	UTI	UTI, pain	pain	–	UTI	–
Postoperative hospital stays, days	10	16	11	10	12	14	12 ± 2
MAP at 2 h after operation, mmHg	89	85	94	85	112	94	93 ± 10
MAP on the first postoperative day, mmHg	87	72	85	78	99	80	84 ± 9
MAP on the second postoperative day, mmHg	94	82	93	84	97	76	88 ± 8
MAP on the third postoperative day, mmHg	87	93	91	88	104	77	90 ± 9
ΔMAP^b^, mmHg	4	− 5	− 3	− 9	− 7	− 5	− 4 ± 4
Urine volume on the first postoperative day, ml	1070	2400	2020	2050	1700	2550	1965 ± 532
ΔUrine volume^c^, ml	220	700	370	250	400	850	465 ± 254
Scr at 24 h after operation, µ mol/L	118	151	100	76	95	108	108 ± 25
Scr at 3 days after the operation, µ mol/L	70	140	95	79	77	62	87 ± 28
Scr at 7 days after the operation, µ mol/L	57	110	91	82	72	58	78 ± 20
Scr at 1 month after the operation, µ mol/L	59	92	88	67	61	59	71 ± 15
Scr at 12 months after the operation, µ mol/L	80	103	84	62	61	65	76 ± 16
ΔScr 1^d^, µ mol/L	9	11	28	6	2	3	10 ± 10
ΔScr 12^e^, µ mol/L	30	22	24	1	2	9	15 ± 12
PI at 1 month after the operation	0.95	1.54	1.23	1.24	1.05	0.99	1.17 ± 0.22
RI at 1 month after the operation	0.57	0.78	0.71	0.7	0.62	0.59	0.66 ± 0.08
Tumor recurrence or metastasis	–	–	graft removal	–	–	–	–
Hospitalization expenses, yuan (RMB)	44,989	54,713	43,612	49,542	49,773	37,715	46,724 ± 5915

^a^*UTI* urinary tract infection

^b^ΔMAP means the variation of MAP on the first postoperative day compared with preoperative time

^c^ΔUrine volume means the variation of 24-h urine volume on the first postoperative day compared with preoperative time

^d^ΔSCr1 means the variation value of SCr at postoperative 1 month compared with preoperative time

^e^ΔSCr12 means the variation value of SCr at postoperative 12 months compared with preoperative time

## Discussion

In this study, six patients successfully underwent 3D laparoscopic nephrectomy combined with bench surgery and autologous transplantation, which achieved tumor resection and renal function preservation without significant intraoperative and postoperative complications, providing a new option for the treatment of patients with highly complex renal tumors. In terms of the choice of surgical access for radical nephrectomy, although Ju et al. 's study suggested that retroperitoneal access could reduce the interference of the intestine to the operation and provide a better intraoperative field of vision, we adopted the transperitoneal access because the kidney extraction incision and autologous kidney transplantation could be classified as the same incision for reducing postoperative pain [19]. During radical nephrectomy, none of the patients reported damage to the renal pedicle vessels and renal pelvic structure. We attribute this primarily to the accurate depth representation provided by the 3D laparoscopic system, which enhances spatial perception, particularly during the separation of the renal pedicle structure and grasping of the edge of clipping tissue. These inherent advantages significantly enhance surgeon confidence and surgical efficiency, while reducing intraoperative complications; these findings align with those observed in Nguyen et al.’s study [20]. The warm ischemia time was controlled within 2 min. This time was similar to the results of Prudhomme et al., who suggested that 3D laparoscopic kidney extraction was significantly superior to 2D laparoscopic system in terms of total operation time (80.9 ± 10.2 vs. 114. 1 ± 32.3 min in 3D and 2D, *p* = 0.0002) and warm ischemia time (1.7 ± 0.6 *vs.* 2.3 ± 0.9 min in 3D and 2D, *p* = 0.02), these findings might be attributed to the improved speed of the 3D laparoscopic system in terms of vessel separation and ligation [21]. The tumor resection and the transplanted kidney repair were all performed after immersion in cryogenic perfusion fluid. Compared with in situ nephrectomy, sufficient operative time is acquired for tumor removal and wound suturing under external cold ischemia conditions, while the operation under direct vision in vitro ensures a favorable negative incision margin and the collection system closure because some small lesions that were not indicated on imaging can be found with the naked eyes [22]. Intraoperative ultrasound can also be used to visualize the kidney to avoid the residual lesion, especially if the tumor is located in the renal portal. Hence, sufficient operative time and an appropriate surgical perspective allow complete tumor removal without damaging the renal pelvis and renal vessels. Although orthotopic autologous kidney transplantation is also performed, the probabilities of postoperative complications and renal graft resection or even loss due to renal artery pseudoaneurysm, renal artery embolism, or renal artery hypoperfusion are higher when compared with ectopic autologous kidney transplantation. Therefore, we adopted ectopic autologous kidney transplantation, which is routinely performed by most surgeons [23]. Autologous kidney transplantation and repair of graft blood vessels before transplantation were performed by experienced surgeons in our hospital. During the operation, an emphasis was placed on anastomosis of the renal blood vessels and ureter with the pelvic blood vessels and bladder, respectively. After anastomosis completion and resuming the blood supply to the kidney, the tension was carefully observed, and the presence of blood infiltration sites and ischemia was confirmed. Consequently, urine was produced from the transplanted kidney after the blood supply was restored, the surface tension and the pulse of the kidney were normal, the local bleeding sites were relieved, and no ischemia was observed. In this study, the duration of cold ischemia was 121 ± 26 min, compared with the kidney obtained from other hospitals, the cold ischemia time is significantly shorter, which helps to protect the function of the transplanted kidney. Although had no impact on graft survival, a prolonged cold ischemia time was associated with more incidence of delayed graft function and lower graft function [24]. After the blood supply was restored, one patient underwent an autologous blood transfusion because of bleeding. This can be due to an incomplete closure of the dorsal side of the anastomosis between the renal artery and the external iliac artery, owing to the deep position of the iliac artery in the patient. No significant bleeding requiring blood transfusion occurred in other patients during or after the surgery, which can also be proved by the fact that there was no significant decrease in MAP after surgery compared with that before surgery. In a study by Janssen, the most common complication was bleeding, with 50% (6/12) of patients requiring intraoperative and postoperative blood transfusions while two patients required temporary hemodialysis, owing to increased surgical trauma of the open procedure [13]. In our study, although all patients experienced a temporary decline in renal function postoperatively, they gradually recovered without any temporary dialysis and the renal function almost returned to preoperative levels 1 month after the surgery, which due to short warm ischemia period and reduced reperfusion injury. In contrast to the temporary decrease in the SCr level, the 24-h urine output of all the patients increased to varying degrees after surgery, which we considered to be due to the increase in the volume of intravenous fluid after surgery as compared with that before surgery. And none of the patients had renal failure requiring dialysis during the follow-up period, and their ultrasound examination showed that the pulsatility and resistance indexes of the transplanted kidney were normal. However, due to the longer lesion resection and wound repair time under cold ischemia, renal cell carcinoma patients experience chronic renal graft failure more frequently than other patients undergoing this surgery for renal aneurysm or ureteral stenosis, which reminds us not to neglect the monitoring of renal function [25, 26]. Although our pathology results indicated that the surgical margins were negative in all the patients, one patient (patient 3) had a recurrence of the transplanted renal tumor 20 months after surgery and underwent transplanted kidney resection in the same month; the postoperative pathology diagnosis was clear cell carcinoma. Therefore, constant monitoring for tumor recurrence is very crucial, even though the other patients had no tumor recurrence and distant metastasis during the follow-up period. Another study by Tran showed that 50% of the patients (4/8) still had tumor recurrence despite negative incisal margins; thus, this might be related to the high aggressiveness of central tumors [27]. Similar to the study by Cowan, we observed that in addition to temporary renal failure, the most common complication was urinary tract infection (4/6), followed by postoperative pain (3/6). In their study, the most common postoperative complications were infection and bleeding, while two patients underwent renal graft resection for renal artery dissection and renal graft failure, respectively, thereby suggesting that cold ischemia time significantly correlates with the incidence of postoperative complications [28]. We suggest that the higher incidence of urinary tract infections is related to a patient’s lower immunity due to the prolonged operation time and postoperative induration of the double-J tube, which can be alleviated after symptomatic antibiotic treatment. With the gradual recovery of patients’ baseline conditions, recurrent and multiple infections did not occur. Although the surgical wound area has been minimized, 50% of the patients still need analgesic drugs to relieve pain after surgery because of the surgical wounds involving both the renal fossa and iliac fossa. Fortunately, a gradual wound repair alleviated long-term chronic pain, and no postoperative incision infection and incisional hernia occurred as in other studies [29, 30]. This is mainly due to the use of 3D laparoscopic nephrectomy, a minimally invasive technique, which reduces the incidence of incision-related complications compared with open nephrectomy [31]. There were no serious postoperative complications in this study, but the monitoring and nursing work of patients during the perioperative period should not be slacken. For some studies have shown that the incidence of postoperative complications such as intestinal obstruction, venous thromboembolism, and pulmonary infection is high, especially for obese patients and patients with chronic renal insufficiency, the incidence of postoperative complications is higher than the general population [12, 32]. The short duration of postoperative hospitalization and the utilization of cost-effective 3D laparoscopic surgery rather than expensive robot-assisted surgery, have effectively maintained the overall expenses associated with this study at a modest level that is affordable for most patients.

The main limitation of this article is the patient population was too small to carry out an effective comparative study with other surgical procedures, so drawing any comparative conclusion was difficult. For example, studies have shown that robotic technology can benefit patients in terms of postoperative complications and recovery time by providing surgeons with a better view of the intra-operative field, enhanced precision and flexibility, and a smaller surgical incision than traditional open surgery [33, 34]. Hence, increasing sample sizes and comparative studies with robotic surgery are future research directions. Furthermore, as this was a single-center retrospective study, our results were greatly influenced by the medical level of the center. Subsequent multicenter studies are needed to evaluate the efficacy and safety of this surgical intervention. Thirdly, limited by the hospital conditions, we solely relied on the SCr level to evaluate the overall renal function in this study. In future research, we will accurately evaluate the differential renal function by radiorenogram or other examinations in other hospitals. In addition, our study’s follow-up time was short, especially for the preservation of transplanted kidney function and tumor recurrence; hence, it is not possible to draw long-term conclusions.

## Conclusion

This study showed that 3D laparoscopic radical nephrectomy combined with bench surgery and autotransplantation is effective and feasible for treating complex renal cancer patients by experienced surgeons. It can be a reliable surgical option for patients with highly complex renal cancer, with a favorable acceptable rate of surgical complications and maximum preservation of renal function. Thus, our study might provide a valid reference for the future development of clinical therapies for highly complex renal tumors.

### Supplementary Information

**Additional file 1: Fig. Supplement**. Three-dimensional laparoscopic nephrectomy. The renal pedicle was fully exposed. The renal artery (a to d) and vein (e to h) were transected after applying Hem-olocks to the clamp.

## Data Availability

The datasets used and/or analyzed during the current study are available from the corresponding author on reasonable request.

## Abbreviations

NSS: Nephron-sparing surgery
RN: Radical nephrectomy
3D: Three dimensional
WIT: Warm ischemia time
SCr: Serum creatinine
R.E.N.A.L: [(R)adius, (E)xophytic/endophytic properties, (N)earness to the collecting system or sinus, (A)nterior (a)/posterior (p) and the (L)ocation]
BMI: Body mass index
MAP: Mean arterial pressure
CT: Computed tomography
CTA: Computed tomography angiography
PI: Pulsatility index
RI: Resistance index
eGFR: Estimated glomerular filtration rate
KIRC: Kidney clear renal carcinoma
UTI: Urinary tract infection

## References

[1] Siegel RL, Miller KD, Jemal A (2020). Cancer statistics, 2020. CA Cancer J Clin.

[2] Hancock SB, Georgiades CS (2016). Kidney Cancer. Cancer J..

[3] Gray RE, Harris GT (2019). Renal cell carcinoma: diagnosis and management. Am Fam Physician.

[4] Gershman B, Thompson RH, Boorjian SA (2018). Radical versus partial nephrectomy for cT1 renal cell carcinoma. Eur Urol.

[5] Bigot P, Hetet JF, Bernhard JC (2014). Nephron-sparing surgery for renal tumors measuring more than 7 cm: morbidity, and functional and oncological outcomes. Clin Genitourin Cancer.

[6] Lombardo R, Leonardo C, Zarraonandia A (2019). Complex renal masses: partial or no partial nephrectomy?. Ann Transl Med..

[7] Kunath F, Schmidt S, Krabbe LM (2017). Partial nephrectomy versus radical nephrectomy for clinical localised renal masses. Cochrane Database Syst Rev.

[8] Campbell SC, Clark PE, Chang SS (2021). Renal mass and localized renal cancer: evaluation, management, and follow-up: AUA Guideline: Part I. J Urol.

[9] Deng W, Zhou Z, Zhong J (2020). Retroperitoneal laparoscopic partial versus radical nephrectomy for large (≥ 4 cm) and anatomically complex renal tumors: a propensity score matching study. Eur J Surg Oncol.

[10] Campbell S, Uzzo RG, Allaf ME (2017). Renal Mass and Localized Renal Cancer: AUA Guideline. J Urol.

[11] Hardy JD (1963). High ureteral injuries. Management by autotransplantation of the kidney. JAMA.

[12] Moghadamyeghaneh Z, Hanna MH, Fazlalizadeh R (2017). A nationwide analysis of kidney autotransplantation. Am Surg.

[13] Janssen MWW, Linxweiler J, Philipps I (2018). Kidney autotransplantation after nephrectomy and work bench surgery as an ultimate approach to nephron-sparing surgery. World J Surg Oncol.

[14] Steffens J, Humke U, Ziegler M (2005). Partial nephrectomy with perfusion cooling for imperative indications: a 24-year experience. BJU Int.

[15] Dirie NI, Wang Q, Wang S (2018). Two-dimensional versus three-dimensional laparoscopic systems in urology: a systematic review and meta-analysis. J Endourol.

[16] Alameddine M, Moghadamyeghaneh Z, Yusufali A (2018). Kidney autotransplantation: between the past and the future. Curr Urol Rep.

[17] Ruan Y, Wang XH, Wang K (2016). Clinical evaluation and technical features of three-dimensional laparoscopic partial nephrectomy with selective segmental artery clamping. World J Urol.

[18] Sánchez-Margallo FM, Durán Rey D, Serrano Pascual Á (2021). Comparative study of the influence of three-dimensional versus two-dimensional urological laparoscopy on surgeons' surgical performance and ergonomics: a systematic review and meta-analysis. J Endourol.

[19] Ju X, Li P, Shao P (2016). Retroperitoneal laparoscopic nephrectomy combined with bench surgery and autotransplantation for renal cell carcinoma in the solitary kidney or tumor involving bilateral kidneys: experience at a single center and technical considerations. Urol Int.

[20] Nguyen DH, Nguyen BH, Van Nong H (2019). Three-dimensional laparoscopy in urology: Initial experience after 100 cases. Asian J Surg.

[21] Prudhomme T, Roumiguié M, Benoit T (2019). Laparoscopy for living donor left nephrectomy: comparison of three-dimensional and two-dimensional vision. Clin Transplant.

[22] Nayak JG, Koulack J, McGregor TB (2014). Laparoscopic nephrectomy, ex vivo partial nephrectomy, and autotransplantation for the treatment of complex renal masses. Case Rep Urol.

[23] Artiles Medina A, Gómez Dos Santos V, Díez Nicolás V (2022). Kidney autotransplantation and orthotopic kidney transplantation: two different approaches for complex cases. Adv Urol..

[24] van de Laar SC, Robb ML, Hogg R (2021). The impact of cold ischaemia time on outcomes of living donor kidney transplantation in the UK living kidney sharing scheme. Ann Surg.

[25] Gwon JG, Kim YH, Han DJ (2017). Real renal function after renal autotransplantation through the analysis of solitary kidney autotransplantation cases. Transplant Proc.

[26] Decaestecker K, Van Parys B, Van Besien J (2018). Robot-assisted kidney autotransplantation: a minimally invasive way to salvage kidneys. Eur Urol Focus.

[27] Tran G, Ramaswamy K, Chi T (2015). Laparoscopic nephrectomy with autotransplantation: safety, efficacy and long-term durability. J Urol.

[28] Cowan NG, Banerji JS, Johnston RB (2015). Renal autotransplantation: 27-year experience at 2 institutions. J Urol.

[29] Crafa F, Rossetti ARR, Striano A (2021). Ex vivo nephron-sparing surgery and kidney autotransplantation for renal tumors. J Surg Case Rep..

[30] Scott T, Venuthurupalli SK (2022). Kidney autotransplantation as a treatment for resistant hypertension due to renal artery stenosis: a case report and review of the literature. Clin Nephrol Case Stud.

[31] Wagenaar S, Nederhoed JH, Hoksbergen AWJ (2017). Minimally invasive, laparoscopic, and robotic-assisted techniques versus open techniques for kidney transplant recipients: a systematic review. Eur Urol.

[32] Vrakas G, Sullivan M (2020). Current Review of Renal Autotransplantation in the UK. Curr Urol Rep.

[33] Kaouk J, Eltemamy M, Aminsharifi A (2021). Initial experience with single-port robotic-assisted kidney transplantation and autotransplantation. Eur Urol.

[34] Pein U, Girndt M, Markau S (2020). Minimally invasive robotic versus conventional open living donor kidney transplantation. World J Urol.

