# Supplementary material for: Clinical experience of bench surgery combined with autotransplantation after three-dimensional laparoscopic nephrectomy for the treatment of highly complex renal tumor
Source: World J Surg Oncol. 2023 Nov 29;21:373. doi: 10.1186/s12957-023-03246-9 (PMC10687882; doi:10.1186/s12957-023-03246-9)
Supplement: Supplementary file 1 — Additional file 1: Fig. Supplement. Three-dimensional laparoscopic nephrectomy. The renal pedicle was fully exposed. The renal artery (a to d) and vein (e to h) were transected after applying Hem-olocks to the clamp. [file 12957_2023_3246_MOESM1_ESM.pdf]

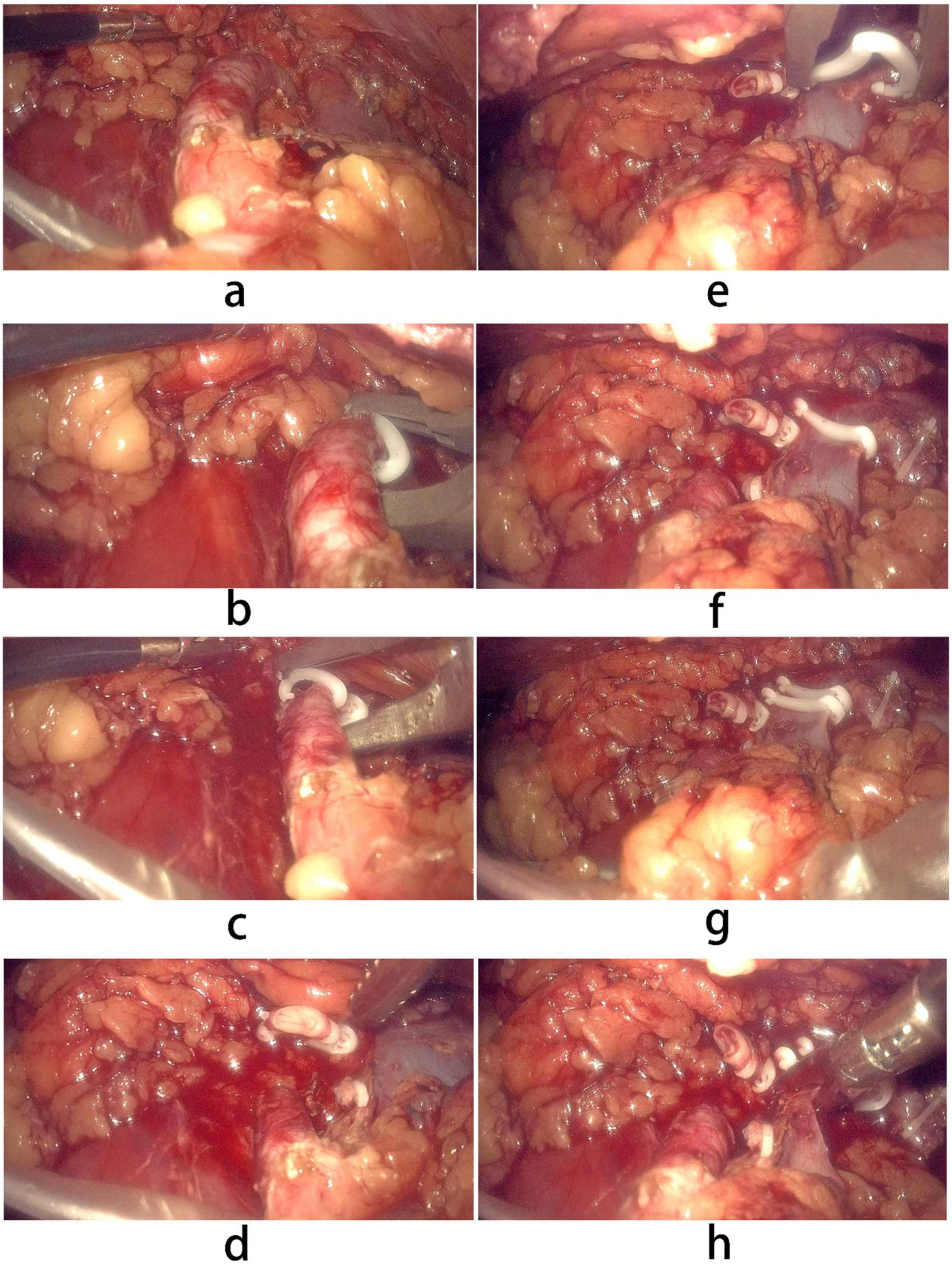

Fig. supplement Three-dimensional laparoscopic nephrectomy. The renal pedicle was fully exposed. The renal artery (a to d) and vein (e to h) were transected after applying Hem-o-locks to the clamp.
